# Financial challenges in the family physician programme in Iran: A systematic review of qualitative research

**DOI:** 10.51866/rv.254

**Published:** 2023-10-11

**Authors:** Abtin Heidarzadeh, Bita Hedayati, Shadrokh Sirous, Mark K. Huntington, Mehdi Alvandi, Alireza Arabi, Babak Farrokhi, Marzieh Nojomi, Somayeh Noori Hekmat, Roksana Mirkazemi

**Affiliations:** 1 BSc, MSc, PhD, Founder and Managing Director of Farzanegan Nik Andish Institute for the Development of Knowledge and Technology, Tehran, Iran. Email: r.mirkazemi@gmail.com; 2 MD, MPH, Medical Education Research Center, Department of Community and Family Medicine. School of Medicine. Guilan University of Medical Sciences, Rasht, Iran.; 3 MPH, Research Department, Farzanegan Nik Andish Institute for the Development of Knowledge and Technology, Tehran, Iran.; 4 MD, National Professional Officer and Unit Head, Universal Health Coverage/Health System, WHO Representative Office, Tehran, Iran.; 5 MD, PhD, FAAFP Professor, Director, Sioux Falls, Family Medicine Residency program and founding Director, Pierre Rural, Family Medicine Residency program, University of South Dakota Sanford, School of Medicine, Vermillion, United States.; 6 MD, Department of Public Administration, Faculty of Management, University of Tehran, Tehran, Iran.; 7 Family Physician Specialist, National Center for Health Insurance, Research, Tehran, Iran.; 8 MD, MPH, Executive Deputy, National Director for Family Medicine, Undersecretary for Health Affairs, Health Network Administration Center, Ministry of Health and Medical Education, Tehran, Iran.; 9 MD, MPH, Preventive Medicine and Public Health Research Center, Psychosocial Health Research Institute, Department of Community and Family Medicine. School of Medicine. Iran University of Medical Sciences, Tehran, Iran.; 10 PhD, Associate Professor of Health Services Management, Leadership and Management in Medical Education Research Center, Kerman University of Medical Sciences, Kerman, Iran.

**Keywords:** Iran, Physicians, Family, Financial management, Systematic review

## Abstract

**Introduction:**

The family physician programme (FPP) was implemented nearly two decades ago as a major health reform. Since the health system and FPP function in a rapidly changing social and economic environment, successful expansion of the programme requires a detailed analysis of its multiple major challenges, including the crucial aspect of its funding system. This systematic review aimed to assess the challenges in the FPP relative to its financing.

**Method:**

All published articles related to the FPP in Iran were included in this study. In particular, original qualitative studies published in English or Persian from 2011 to 2021 were included. In January 2022, international credible scholarly databases and Persian databases were searched. All selected articles were carefully studied, and the data were extracted using the sample, phenomenon of interest, design, evaluation and research type technique. The Preferred Reporting Items for Systematic Reviews and Meta-Analyses were used in preparing the study report.

**Results:**

Among 491 articles retrieved from the search strategy, 50 met the inclusion criteria after their titles and abstracts were screened. Twenty-nine studies were excluded after their full texts were reviewed. A total of 11 eligible empirical studies were finally included. Based on the results, six broad categories (budget and funding, insurance system, tariffs, payments, accountability and injustice) were identified as financial challenges.

**Conclusion:**

This study identified the challenges associated with financing among family physicians, and the results could provide guidance for policy-making in the expansion of the FPP

## Introduction

According to the World Health Organization (WHO), financing is one of the six building blocks of health care systems.^1,2^ It is also the main pillar among the ‘necessary features of the national health system’ and at the heart of its success.^3^

Health system finance is a long-standing challenge in Iran, with a high rate of out-ofpocket payments.^4,5^ The distribution of health expenditure in Iran in 2008 was as follows: 24.9% paid by the general governmental budget, 20% paid by social health insurance, 52.7% paid out of pocket and 2.4% paid by other private sources.^6^ In the same year, urban and rural households paid in average 6.4% and 6.35% of their total expenditure on health services, respectively.^7^ While social protection measures and insurances reduce the pressure on household welfare, the inadequate targeting of benefits and the lack of correlation of their value with inflation have decreased their impact over time.^7^ Hajizadeh and Nghiem suggested that a single universal health insurance plan can save households from catastrophic health spending despite different employment status.^8^

Iran experienced different reforms in its health care system to increase accessibility to health services. The country implemented the family physician programme (FPP) nearly two decades ago^9^ as a major health reform.^10^

The initial plan was to establish the programme in four provinces in Iran and then expand it to other provinces. At the beginning of the implementation process, some modest achievements were reported^11^; however, the FPP was not expanded owing to multiple challenges.

Among the challenges in the FPP in Iran are financial and insurance issues.^12,13^ The proximal sources of programme fUnding are taxation and insurance premiums.^3^

The FPP in Iran was supposed to be sourced through the governmental budget. The required budget ‘was foreseen and approved in the budget law’. However, the allocation of the budget was a matter of concern.^14^

While the successful implementation of the FPP closely relied on insurance organisations, studies have shown that insurance companies were not ready to embrace the FPP owing to hasty initiation of the programme without addressing the required infrastructures.^15^ This scenario worsened because of the unsatisfactory operational history of insurance organisations, such as their long-overdue debts to health care providers.^16^

The health system and FPP function in a rapidly changing social and economic environment, and successful expansion of the programme requires a detailed analysis of its multiple major challenges, including the crucial aspect of its funding system.^17,18^ Since the introduction of the FPP, many studies have evaluated the programme from various dimensions, including its financial aspect. This study aimed to synthesise data from these studies via a systematic review to obtain comprehensive results. In particular, this systematic review was undertaken to identify the main challenges in the FPP relative to its financing.

## Methods

The study protocol was developed by the authors. The methodology for publication selection and retrieval as well as data extraction and synthesis is described below.

### Eligibility criteria

All published articles related to the FPP in Iran were included in this study. In particular, original qualitative studies published in English or Persian from 2011 to 2021 related to financing of the FPP were included. Conversely, grey literature, quantitative studies, systematic reviews, commentaries, editorials, case reports, cross-sectional studies and studies published in languages other than English or Persian were excluded.

### Information sources

In January 2022, international credible scholarly databases (Google Scholar and PubMed) and Persian databases (Iran Medex, Magiran, Irandoc and SID) were searched. In addition, the references of the selected articles were manually searched to find additional relevant studies.

### Search strategy

The search strategy was defined based on keywords and the search syntax, which was first defined for the PubMed database and then revised based on each database’s specific framework of search method.

The following keywords were used in both English and Persian: ‘family physician’, ‘family physician care program’, ‘general practice’, ‘general medicine’, ‘general practitioner’, ‘general physician’, ‘insurance’, ‘finance’, ‘budget’, ‘fund’, ‘coverage’, ‘tariffs’, ‘salary’, ‘wage’, ‘payment’, ‘per capita payment’, ‘performance-based payment’ and ‘Iran’. These keywords were employed individually and in combination using the Boolean operators ‘AND’ and ‘OR’.

### Selection process

Based on the title and abstract of the articles, two reviewers independently evaluated the articles returned by the search in accordance with the inclusion criteria. Duplicate articles were removed at this stage. The studies were classified into three categories: ‘excluded’, ‘included’ or ‘probable’. The reviewers then evaluated the full text of the articles categorised as ‘probable’ and re-assigned them to either ‘included’ or ‘excluded’. The lists generated by the reviewers were compared, and articles for which both reviewers agreed on categorisation were either excluded or included. When there was disagreement between the reviewers’ assigned category of articles, the disputed articles were either included or excluded based on the evaluation by a third reviewer.

### Data collection process

All selected articles were carefully studied, and the following data were extracted: title, authors, year of publication, name of the journal, design, participants, instruments, settings, variables, strengths and weaknesses.

For the final review, all selected articles were carefully studied. As this study was a systematic review of qualitative studies, qualitative techniques of data extraction, including the sample, phenomenon of interest, design, evaluation and research type (SPIDER) technique, were used.^19^ The Preferred Reporting Items for Systematic Reviews and MetaAnalyses were used in preparing the report of this study.

After the full-text review, studies that lacked the aforementioned data to be extracted using the SPIDER technique were excluded from the analysis.

### Data items

Challenges related to the insurance and financial aspects of the FPP in Iran were the data items in this study.

### Risk of bias assessment

Two independent reviewers conducted the eligibility and quality assessments and data extraction and sought the opinion of a third reviewer in cases of a difference in opinion.

A methodologist checked the validity of the studies in accordance with the international guidelines for reporting of research, such as the Consolidated Criteria for Reporting Qualitative Studies. Published articles with low validity were excluded from the study.

### Data synthesis

A thematic synthesis method was used to synthesise qualitative data from the included studies. The synthesis process consisted of three interconnected stages. Multiple readings of the studies were conducted to ensure comprehensive coverage of views from the studies. First, the primary study findings were coded line by line. Second, the codes were organised into categories that were related, leading to the development of descriptive themes. Third, analytical themes were extracted from the categorised codes. Differences and similarities were examined, leading to the creation of a thematic structure by grouping emerging concepts. The final stage yielded six detailed analytical themes.

## Results

**Figure 1:**
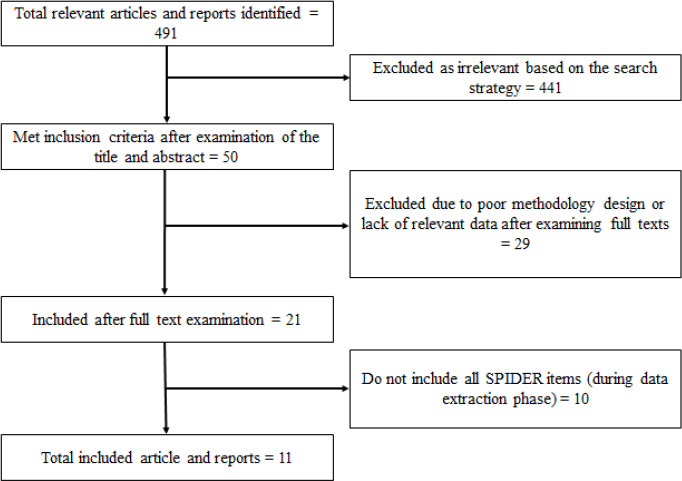
Flowchart of the publication selection.

A total of 491 articles were retrieved from the search strategy, including 488 from the database search and three from the manual search. Of the 491 retrieved articles, 441 were excluded: 22 owing to duplication and 419 owing to irrelevance to the research strategy. Fifty studies met the inclusion criteria after screening of the titles and abstracts. After the full-text review, 29 studies were excluded owing to either a poor methodology design or a lack of data relevant to the research questions. Ultimately, a total of 11 eligible empirical studies were included in the present review (Figure 1)

### Study characteristics

Table 1 shows the characteristics of the 11 included studies. All 11 studies used qualitative methods; the data were collected via interviews and focus group discussions. A total of 277 interviews and 29 focus group discussions were conducted in these studies.

**Table 1:** Characteristics of the included studies.

Author	Publication year	Data collection	Participants	Sample size	Location	Urban/rural family physician
Doshmangir et al.^16^	2017	Interview	Informed individuals from the Ministry of Health, health insurance organisations, management and planning organisation in Iran, the Iran Medical Council, medical universities and health research centres	19	Cities in Iran	Urban
Fardid et al.^20^	2019	Interview and focus group discussion	National and regional policy-makers, managers, physicians, patients, health professionals and FPP officers who influenced the decision-making process and FPP implementation	24 (interview) and 3 (focus group discussion)	Fars province	Urban
Mehrolhassani et al.^14^	2021	Interview	Policy-makers and managers at national and provincial Levels	44	Kerman province	Urban
Dehnavieh et al.^15^	2015	Interview	Informed individuals from medical universities, health service insurances and medical systems as well as social physicians and researchers in the field of family medicine	21	Cities in Kerman province	Urban
Gharibi and Dadgar^21^	2020					
Mohammadi Bolbanabad et al.^22^	2019	Interview and focus group discussion	Managers, experts, family physicians, specialists, midwives, health insurance experts, service recipients and Behvarz	30 (interview) and 5 (focus group discussion)	Kordestan province	Rural
Abedi et al.^23^	2017	Interview	Family physicians, senior managers, experts and board members	9	Iran	Urban
Hooshmand et al.^24^	2020	Interview	Managers and family physicians	20	Khorasan Razavi province	Rural
Alaie et al.^25^	2020	Interview	Policy-makers and informants	26	Iran	Urban/rural
Farzad et al.^26^	2018	Interview and focus group discussion	Family physicians, midwives, managers, health insurance managers and service recipients	37 (interview) and 21 (focus group discussion)	Kordestan, Alborz and West Azerbaijan provinces	Urban
Kaskaldareh et al.^27^	2021	Interview	Health network assistants, health network development authorities,	15	Gilan province	Urban/rural

The participants included family physicians and other specialists, policy-makers, managers, nonphysician health professionals such as midwives and Behvarz (community health workers) and patients. Individuals from the Ministry of Health, health insurance organisations, management and planning organisations in Iran, the Iran Medical Council, medical universities and health research centres as well as social physicians and researchers in the field of family medicine were also among the participants.

Six of the eleven studies investigated the urban FPP; two studies, the rural FPP; and three studies, both urban and rural FPPs.

Table 2 shows the main findings of the 11 studies regarding the insurance and financial challenges in the FPP, while Table 3 summarises these challenges into six broad categories: budget and funding, insurance system, tariffs, payments, financial accountability and inequity.

**Table 2:** The insurance and financial challenges in the Family Physician Program (FPP) of Iran based on systematic review of the publications

Author (year)	Insurance and financial challenges
Doshmangir et al. (2017)^14^	Unreadiness of the current health insurance system to embrace a great health system reform such as the FPP Unsatisfactory health insurance schemes in the past Long-term liabilities of health schemes to health care providers Delayed reimbursements by health insurances to family physicians Not fulfilling insurance obligation regarding the FPP Lack of pooled fund and fragmented health insurance system Lack of a public insurance scheme No rational medical tariffs based on the relative value of health services Inadequate financial resources, underestimation of the required funds for the plan, allocation of available financial support by entities other than the responsible institutions and lack of clear and stable financial resources for the programme
Fardid et al. (2019)^20^	Multiple insurance funds Delayed payments to family physicians Spending of the allocated budget for other purposes
Mehrolhassani et al. (2021)^14^	Different insurance organisations and policies (different policies in health care and social security insurances and different insurances, such as oil industry or banks) The FPP in other countries has either followed the national health system or integrated insurance funds, but Iran has followed neither. Coordination between family physicians and insurance companies was later facilitated at provincial and local levels, so physicians began accepting their insurance cover. Payment and service purchase system: ‘per capita^1^’ payment to family physicians and their teams versus ‘single payment’ for levels 2 and 3^2^ Long delay (years) in payment of the approved budget (one of the reasons was the change in ministers and governments over the years) Some governments did not have a plan to allocate the budget to the FPP, so it was postponed.
Dehnavieh et al. (2015)^15^	Initiating the FPP before integrating the insurance schemes Lack of backup software for payment methods Unclear methods of payment Different payment methods for different suppliers Difficulties in supervision owing to multiple payment methods Delayed payments (through an intermediary) Insufficient financial resources Weak financial processes Unpredictable economic conditions and sanctions imposed on Iran led to additional financial problems for the FPP Injustice in funding
Gharibi and Dadgar (2020)^21^	Insufficient infrastructure for a performance-based payment system (value-based payment) Individual-centred payments instead of salaries Clinical and treatment approaches of managers who pay family physicians Lack of criteria and scientific tools for qualitative assessment of the FPP for a performance-based payment (no direct relationship between performance indicators and the amount of effort by physicians; different work conditions were not considered in the monitoring process; subjective monitoring was used instead of the objective type) A top-down monitoring approach instead of an educational approach, can enhance the FPP Lack of trained and experienced assessors for the FPP Low primary health care budget relative to hospital services (discouraging qualified individuals from participating in the FPP, decreasing primary health care related interventions, and paying insufficient attention to the priority of prevention by health insurance systems) Clinical and treatment approaches of managers who pay family physicians Low wages of family physicians compared with those of specialists Clinical and treatment views of managers in charge of paying family physicians
Mohammadi Bolbanabad et al. (2019)^22^	Insurance deductibles Lack of a health-oriented vision of insurance Lack of a proper supervision structure in the health insurance organisation to monitor the FPP and rural insurance Delay in payment Insurance deductibles make it difficult to provide equipment for rural health service centres and houses. Delayed budgeting
Abedi et al. (2017)^23^	Using a per capita model instead of a function-based model for payments to health care teams Lack of a health-oriented vision of insurance organisations Insurance inspectors do not have the expertise to assess the performance of physicians No training programme for insurance inspectors No wage specified for insurance inspectors monitoring the FPP No monitoring by insurance inspectors during afternoon hours Monitoring by insurance organisations was conducted to determine family physicians’ salary, not to assess the progress towards the FPP goals. Unjust payment to members of family physician teams Difference in tariffs set for urban and rural physicians Delayed payment Multiple insurance funds and lack of coordination between them Discrimination between those insured by social security and health insurance organisations and those insured by other organisations to access physicians
Hooshmand et al.(2020)^24^	Lack of valid and reliable checklists for FPP assessment Lack of inspectors with the required expertise to evaluate family physicians’ performance High insurance deductibles and delayed income definition Inadequate criteria for per capita income definition Low determined per capita of the target population (not considering foreigners in determining per capita ( Incomplete service package Ignoring social and cultural conditions while localising service packages Disproportionate service package relative to the target population size Inadequate salaries Ignoring inflation, educational level and hardship level in determining salaries Lack of incentive payments and less motivation for specialists to participate in the family physician plan
Alaie et al. (2020)^25^	Inadequate budget for insurance schemes Lack of a national policy for tariff Failure to provide the necessary budget
Farzad et al. (2018)^26^	Unclear way of payments (It is not clear to family physicians how much they will receive for the service) Delayed payments Lack of inspectors with the required expertise to evaluate family physicians’ performance Incomplete service package Providing free service packages (people’s referrals and the expectations for additional medication and clinical testing will increase) Not integrating existing insurance schemes Problem in providing budget Injustice in payments Lack of incentive payments and less motivation for specialists to participate in the family physician plan
Kaskaldareh et al. (2021)^27^	Lack of budget Unclear payment methods

**Table 3:** Insurance and financial challenges in the Family Physician Program (FPP) of Iran based on systematic review of the publications

Theme	Subtheme
Budget and funding	Lack of clear and stable financial resources for the FPP
	Injustice in funding
	Insufficient financial resources
	Delayed budgeting
	Underestimation of the required funds for the plan
	Spending the allocated budget for other purposes
	Weak financial processes
	Lack of a pooled fund
Insurance system	Multiple policies, fragmented health insurance system and lack of coordination among insurance organisations
	Lack of a public insurance scheme
Tariffs	No rational medical tariffs based on the relative value of health services, inflation, educational level and hardship of work
	Lack of a national policy for tariff
	Payment per capita instead of performance-based payment to health teams
Payments	Single payment for levels 2 and 3
	Insufficient infrastructure for a performance-based payment system
	Inadequate criteria for per capita payment
	Individual-centred payments instead of salaries
	Clinical and treatment approaches of managers who pay family physicians
	Lack of a health-oriented vision of insurance organisations
	Delayed reimbursements by health insurances to family physicians
	Unclear methods of payment
	Lack of a backup software for methods of payment
	High insurance deductibles
Financial accountability	Lack of trained and experienced inspectors with the required expertise to evaluate the FPP
	Lack of proper supervision structure in health insurance organisations
	Difficulties in supervision owing to diverse payment methods in the FPP
	Lack of criteria and scientific tools, such as valid and reliable checklists for FPP assessment
	Top-down monitoring approach instead of an educational approach, which can enhance the FPP
Inequity	Unjust payments to members of family physician teams
	Difference in tariffs set for urban and rural physicians
	Lower wages for family physicians than for other medical specialists
	Discrimination between those insured by social security and health insurance organisations and those insured by other organisations to access physicians

### Budget and funding

Nine studies addressed the challenges related to budgeting. There was a lack of clear and stable financial resources for the FPP. This challenge was seen in the inequity of funding, insufficient financial resources due to underestimation of the funds required for the plan, delayed disbursement of funds, diversion of the allocated budget to other purposes and weak financial processes.^14-16,20-22,25-27^

Financial resource allocation was a particular challenge, with lower budgets allotted to primary care services than to hospital-based services. This undermined the FPP by discouraging qualified individuals from participating in the FPP, decreasing the implementation of PHC-related interventions and resulting in insufficient attention to the priority of prevention by the health insurance system.^21^

### Insurance system

The lack of a pooled fund and the fragmented health insurance system were the main challenges in this area.^14,16,20,23,26^ The FPP was implemented prior to integration with insurance; coordination between the FPP and insurance companies was later initiated at the provincial and local levels.^14,15^ There was no coordination between multiple insurance policies and organisations and other payer sources, such as Iran Health Insurance, social security organisation, oil industry, banks and armed force.^14,20,23,26^ One study suggested merging social health insurance funds and establishing the Iran Health Insurance Organization as a proposed single fund.^16^ While the implementation of the FPP required public insurance, the studies showed that there is currently no effective public insurance in the country.^16^

### Tariffs

Tariffs, the fees charged to patients for health care services, are another challenge encountered in the FPP. The lack of a national policy for tariffs and the absence of rational medical tariffs based on the relative value of health services, taking into account patients’ ability to pay, were among the identified challenges.^16,24,25^

### Payments

Per capita payment to family physicians and their teams compared with single payment to specialists at the secondary and tertiary levels of health care systems was another financial challenge related to the payment methods.^14,23^ While performance-based payment was considered the most appropriate payment method for the FPP, there was insufficient infrastructure to implement such method in Iran.21 The population upon which the per capita payment was based for physicians was not well defined, and the per capita payment amounts were low, presenting additional challenges in this area.^24^

Contrary to the intent of the FPP, a curative rather than a preventative focus by managers who pay family physicians and a similar lack of a population health-oriented vision by insurance organisations led to the employment of exclusively treatment-focussed measures in determining payments.^21-24^

Delayed reimbursements by health insurances to family physicians and their teams, unclear methods of payment and lack of a backup software for methods of payment were among the insurance and financial challenges noted. It was unclear to family physicians when or how much they will receive for the provided services, further discouraging participation in the programme.^15,16,20,22-24,26,27^

### Financial accountability

Another challenge related to FPP financing was inadequate oversight. There was no adequate supervisory structure within health insurance organisations for monitoring the FPP and rural insurance payments^22^; the existence of multiple payment methods greatly complicated the task of oversight.^15^

There was a lack of trained and experienced assessors in the programme: Inspectors did not have the required expertise to assess the performance of family physicians. There was no provision for funding of inspections in the programme.^21,23,24,26^

Criteria and scientific tools for qualitative assessment of the FPP for performance-based payment were lacking. There were no valid and reliable checklists for FPP assessment; in the absence of defined measures, monitoring was conducted subjectively.^21,24^ Differences in local conditions such as available resources or burden of a disease in a population were not taken into consideration during the monitoring process.

Using top-down and investigative-like approaches for monitoring instead of a collegial approach for enhancing the FPP was another challenge encountered. The approach was perceived as punitive rather than constructive. Monitoring by insurance organisations was conducted to determine the salary of individual family physicians, not to assess the achievement of the broader goals of the FPP.^21,23^

The availability of inspectors during relevant times was deficient. Insurance offices were closed in the afternoon, so family physicians were not monitored during afternoon hours, during which time most of the work is done.^23^

### Inequity

Discrimination was another challenge noted. Differences in reimbursement sets between urban and rural family physicians, disparate salaries of members of family physician teams, lower wages for family physicians than for other physicians and discrimination in access to physician care between those insured by social security and health insurance organisations and those insured by other organisations were among the identified problems.^21,23,24,26^ The absence of incentive payments resulted in low motivation for specialists to participate in the FPP.^24,26^

## Discussion

Nearly two decades since the introduction of the FPP in Iran, many challenges have been identified, which explain why the programme has failed to expand beyond the four initial provinces.^9,15,20,28-31^ One of the main identified challenges was related to the financial and payment systems,^12,14-16,20-27^ which this systematic review attempted to address.

In general, the Iran health system experiences many challenges regarding funding and finance.^32,33^ Moghaddam et al. reported inadequate overall and public finances, unsustainable resources and lack of cohesion in stewardship of financing systems as some of the identified challenges.^33^ Adding the FPP imposed more challenges to the existing challenges of an already problematic system. The FPP in other countries has either employed a publicly funded national health service or incorporated private insurance funds, but Iran has followed neither.^14^ One of the reasons identified for the insufficient budget in Iran was the unpredictable economic conditions and financial sanctions in the country.^15^ Further, transitions of governments resulted in inconsistent funding for the FPP, as some administrations did not intend to fund the programme.^14^

Insurance in Iran is fragmented, including multiple policies, lack of coordination among insurance organisations and lack of a public insurance scheme. This challenge has long been recognised. Bazyar et al. reported that in Iran, multiple health insurance funds exist, without adequate provisions for transfer or redistribution of cross-subsidy among them.^34^ Multiple risk pools resulted in inequitable benefits, inefficiency, low financial protection for insured persons, high coinsurance rates, duplication in insurance coverage, discriminated benefit package of public health insurance schemes, underfunding and severe financial shortages of public funds, and lack of transparency and reliable data. A lack of a profound vision in medical insurance and insurance funds with different methods of calculating premiums and collecting revenues was reported by Moghaddam et al.^33^

Fragmentation was also noted in the leadership and management of the FPP. The Ministry of Health and Medical Education is the main policy-maker and is responsible for providing health care services, while the Ministry of Co-Operatives, Labor, and Social Welfare supervises the various public insurance schemes.^35^ These ministries do not commonly work effectively together to achieve their mutual goal of health for the public.

This review also found systematic and non-systematic challenges in the payment system. The payment system for the FPP is per capita and not performance-based. Conversely, the single-payment system is used for secondary and tertiary care levels. Herein, unclear methods of payment because of inadequate criteria for per capita payment, individualcentred payments instead of salary payment, insufficient infrastructure for a performance-based payment system, and lack of backup software for methods of payment were among the main challenges noted. Pay-for-performance schemes have been introduced in some countries to improve the quality of care provided.^37,38^ This approach also has a positive impact on decreasing induced demand and costs.^3^ The per capita payment method has been considered a source of conflict of interest between family physicians and specialists at the secondary care level, who receive performance-based payments.^3^

Delayed reimbursements by health insurances to family physicians were also mentioned as one of the challenges in the payment system herein. Some studies mentioned that payment through an intermediary could be one of the causes of delayed reimbursements.^15,16,20,22,24,26,27^ Further, untargeted health sector resources towards low-income deciles have long been a challenge in financial and payment systems in the Iran health system.^33^

Supervision of the finance and payment system was another challenge. The lack of trained and experienced inspectors with the required expertise to evaluate the FPP, lack of criteria and scientific tools (e.g. a valid and reliable checklist for FPP assessment), and performance-based payment as well as diverse payment methods were some of the reasons behind challenges in supervision. Other challenges were the lack of proper supervision structure in health insurance organisations and a top-down monitoring approach instead of an educational approach, which can enhance the FPP.

Unfair and unjust payments as well as differences in tariffs set for urban and rural physicians, lower wages for family physicians than for other medical specialists such as paediatric or internal medicine physicians, and discrimination between those insured by social security and health insurance organisations and those insured by other organisations to access physicians were some of the issues related to unfair payment and budgeting system in this review. Many studies reported that inequity and unfairness in health financing exist in the health system of the country, affecting the FPP funding.^33,39,40^ Moghaddam et al. showed that the finance system experiences inequity because of ‘equal payment to services with different quality, different prices for a similar service, not to obey the public and private tariffs’.^33^

Tariffs were also another challenging area noted in this review. Tariffs do not account for the relative value of health services, inflation, educational level, or medical complexity. There is no national policy for tariff and payment per capita instead of performance-based payment to health teams. Setting tariffs for health care services has long been recognised as a challenge. This aspect has been sporadic and not evidence-based, resulting in disparity, lack of clarity, conflict of interest, and corruption.^36^

### Potential biases

The present systematic review might have selection bias. In general, selection bias can arise when review authors unintentionally exclude relevant studies or include nonrelevant studies. To address this problem, two reviewers selected the studies in this review.

## Conclusion

This study identified major challenges in different aspects of FPP financing including budget and funding, insurance system, tariffs, payments, supervision of finance, and inequity in the system. These challenges should be addressed prior to any attempt to expand the programme across Iran.

### Recommendations

**Implications for practice**

- A sufficient, clear, and stable financial resource for the FPP is recommended.
- An integrated public insurance is suggested for the FPP.
- A national policy for tariffs that incorporates rational medical pricing, considering both the relative value of health services and patients’ financial capacity to pay, must be established.
- Establishing an infrastructure for performance-based payment is recommended for the FPP
- Insurance organisations should adopt a population health-oriented vision. This change is necessary to avoid relying solely on treatment-based approaches when determining payments.
- It is recommended to address the challenge of inadequate oversight in the financing of the FPP. A crucial step is to establish a robust supervisory structure within health insurance organisations to effectively monitor both FPP and rural insurance payment systems. This oversight mechanism will ensure greater accountability and transparency in the financial management of the programme, leading to improved efficiency and effectiveness in delivering healthcare services.

**Implications for research**

Systematic research on the financial aspects of the FPP, including its cost-effectiveness, and the impact of any intervention on this programme is recommended.

## Review protocol registration

The study protocol was reviewed and approved by the Research Board of Guilan University of Medical Sciences.
